# Supplementary material for: Targeting of SUMOylation leads to cBAF complex stabilization and disruption of the SS18::SSX transcriptome in Synovial Sarcoma
Source: Res Sq. 2024 Jun 6:rs.3.rs-4362092. Preprint. [Version 1] doi: 10.21203/rs.3.rs-4362092/v1 (PMC11177989; doi:10.21203/rs.3.rs-4362092/v1)
Supplement: Supplement 1 [file NIHPPrs4362092v1-supplement-1.pdf]

Supplementary Files

This is a list of supplementary files associated with this preprint. Click to download.

- SUPNC.pdf
- NCOMMS2429445TEditorialNote.docx
